# Exogenous Nitric Oxide and Silicon Applications Alleviate Water Stress in Apricots

**DOI:** 10.3390/life12091454

**Published:** 2022-09-19

**Authors:** Asuman Gundogdu Bakır, Ibrahim Bolat, Kubra Korkmaz, Md. Mahadi Hasan, Ozkan Kaya

**Affiliations:** 1 Ministry of Agriculture and Forestry, Apricot Research Institute, Malatya 44090, Turkey; 2 Department of Horticulture, Faculty of Agriculture, Harran University, Sanliurfa 63050, Turkey; 3 Department of Horticulture, Graduate School of Natural and Applied Sciences, Harran University, Sanliurfa 63050, Turkey; 4 State Key Laboratory of Grassland Agro-Ecosystems, College of Ecology, Lanzhou University, Lanzhou 730050, China; 5 Erzincan Horticultural Research Institute, Erzincan 24060, Turkey

**Keywords:** leaf temperature, malondialdehyde, membrane permeability, proline, stomatal conductivity

## Abstract

Many plants confront several environmental stresses including heat, water stress, drought, salinity and high-metal concentrations that are crucial in defining plant productivity at different stages of their life cycle. Nitric oxide (NO) and Silicon (Si) are very effective molecules related in most of them and in varied biochemical events that have proven to be protective during cellular injury caused by some stress conditions like water stress. In the current work, we studied the effect of Si and NO alone and NO + Si interactive application on the response of plants exposed to water deficiency and well-watered plants for the Milord apricot variety. Water stress caused a reduce in chlorophyll content, dry and fresh weight, leaf area, stomatal conductivity, leaf relative water content and nutrient elements, while it caused an increase in leaf temperature, leaf proline, leaf malondialdehyde (MDA) content and membrane permeability. Si, NO and Si + NO combination treatments under water stress conditions significantly decreased the adverse effects of water stress on leaf temperature, leaf area, dry and fresh weight, stomata conductivity, relative water content, membrane permeability, L-proline and MDA content. The shoot dry weight, chlorophyll content, stomata conductivity and leaf relative water content in Si + NO treated apricot saplings increased by 59%, 55%, 12% and 8%, respectively. Combined treatment (Si + NO) was detected to be more effective than single applications (Si or NO) on some physiological, biochemical, morphological and nutritional properties of apricot under water stress conditions.

## 1. Introduction

Global climate change is one of the hottest issues of the last decade, and it has been emphasized as the most important environmental problem affecting the expansion or contraction of habitats, survival, development and reproduction of many plant species worldwide [1]. Drought brought about by climate change and discussed frequently recently is one of the most destructive abiotic stresses that limit many plant growth, development and yield, especially in semi-arid and arid climates. Some recent studies have, indeed, debated the issue of how certain perennial plants respond to drought stress [1,2,3]. Therefore, increasing drought stress tolerance in plants or accessing information on their drought response mechanisms is vital both to predict potential adaptation pathways and to formulate sensible conservation strategies that can cope with drought stress [2,3]. Basically, various plant biological processes in the cytosol and extracellular matrices, namely, photosynthesis, reactive oxygen species (ROS), respiration rate, mineral nutrition, turgor pressure, carbon assimilation rate and gas exchange properties of leaves, are disturbed by water or drought stress, ultimately causing disruptions in plant life mechanism [1,2,3]. Therefore, underlying the vital research question posed against the backdrop of drought stress begins with understanding how plants perceive signals under stress conditions and how they respond in the event of a problem. When plants encounter drought stress or water deficit, some mechanisms are developed by them, such as tolerance of stress conditions by maintaining the turgor of cells in tissues through osmotic adjustment that allows plants to maintain growth, their escape from stress conditions with rapid development allowing them to complete their cycle before death (i.e., decreasing transpiration rate of plant by the reduction of leaf area and stomata conductance of plant and increasing water uptake), and resisting severe water stress conditions with the aid of various survival mechanisms are adopted by plants [4,5,6].

Recently, the treatments of NO fertilization as an alternative method in plant development in drought stress is becoming increasingly important [1]. Plant responses to NO applications were first described in the 1970s and was identified as a defense signal within the plant in 1998 [2]. Nowadays, it has been highlighted that NO is a messenger molecule that responds to stress in plants under biotic and abiotic stress conditions, and acts as both an antioxidant and an anti-stress agent [2]. In drought stress, NO has been found to provide stress tolerance by regulating the opening and closing of the stoma [3]. In addition, NO is known to act as plant responses against various abiotic and biotic stresses, namely, injury, drought, infection, high and low temperatures, ozone and ultraviolet (UV) in some plants [2]. The complex structure of the drought response plays a key role in the slow progress of the drought recovery studies [4]. Many alternative methods have been investigated to decrease the effect of water deficit stress and increase plant tolerance [5]. Mulching and microorganism applications as well as some treatments such as humic acid, oxalic acid, salicylic acid, Si and NO are among these methods [6]. These practices cause an increase in plant growth and water utilize efficiency [7]. Si has a favorable effect on plant development in plant nutrition and provides tolerance to plants against abiotic stresses [8]. Si in plant tissues provides nutrient uptake from the soil, and thus helps to increase growth by developing tolerance against negativities such as salinity, drought, metal toxicity, nutrient deficiency, pathogens and insects [9]. In drought stress, Si stores a high level of water potential in leaves of plant; furthermore, it increases drought tolerance in plants by raising the amount of endodermal tissue that plays a key role in carrying water to the root [7]. In addition, it reduces sweating and ensures the preservation of water within the plant by reducing the accumulation of salt in Si calcareous soils [10].

Apricot, which is among the deciduous species, is an important species widely grown in Mediterranean countries, although it is cultivated in many parts of the world. In these countries, it has become an indispensable product for producers as it provides a good source of economic income. Although apricot is known as a drought tolerant plant species due to some of its characteristics, water shortage is the biggest problem in the Mediterranean climate zone. Therefore, irrigation is needed for commercial apricot production [11]. In recent years, apricot cultivation has started in many regions of the world under limited water conditions and in waterless areas [12]. The supply of irrigation water needed in agricultural activities is becoming increasingly difficult due to global climate change. Drought stress causes significant negativities in many plant species if the necessary water cannot be provided in a short time. Although apricot is tolerant in arid conditions, it causes some problems to the plant in terms of morphological, physiological, biochemical and molecular in different development periods. As a result, it causes significant losses in terms of quantity and quality [13]. Therefore, one of the most important goals of growers is to develop plant species that can better withstand water stress in different plant growth periods in semi-arid areas with water problems or to increase tolerance to water stress with various mineral applications [14,15].

To the best of our knowledge, there are no previous studies on alleviating drought stress in plants by applying NO, Si and their combination together (Si + NO) [2,7], except to Myrobalan 29 C rootstock [8]. Those studies have, however, addressed the ameliorative effects of Si and NO alone on water stressed plants including tomato, wheat and rice [2,7]. On the other hand, Bolat et al. [8] previously reported that Myrobalan 29 C rootstock effects were significant against water stress conditions; however, it is known that the effect of rootstock alone is not sufficient under stress conditions. As a matter of fact, it is extremely important to consider separately the effects of rootstock, variety and combination of variety x rootstock under water stress conditions. Therefore, each scion–rootstock combination rather than each separate rootstock and scion must be assessed under water stress conditions due to the responses of grafted apricot plants to water stress may vary according to the interaction of scion, rootstock and rootstock–scion. We have also hypothesized the roles of Si, NO and their combination in increasing water stress tolerance in apricot grafted on Myrobalan 29 C rootstock and maintaining sapling growth. The findings noted here provide new data on investigating the role of the three applications (i.e., NO, Si and Si + NO) in mitigation water stress-induced injury and improving water stress tolerance in apricot by the action of three applications on biochemical, physiological, morphological and macronutrient element attributes.

## 2. Materials and Methods

### 2.1. Plant Material and Applications

The current work was carried out at the Research Center of the Harran University of Agriculture Faculty, Department of Horticulture in 2019. Milord apricot variety grafted on Myrobalan 29 C rootstock was utilized as material. Milord apricot variety was bred by PSB production vegetal in Spain. It has a strong, semi-spreading tree structure. Homogeneous one-year-saplings of the Milord apricot variety grafted on Myrobalan 29 C rootstock were planted in 12 L plastic pots containing peat (Klasmann TS1) in mid-March. Plants were cut three nodes above grafting point. In the experiment, peat growing medium with pH 6.5 and N:P:K at 1.0 kg/m^3^ in the ratio 14:10:18 and with sodium silicate (Si) and nitric oxide (NO) donor sodium nitroprusside (SNP) solution were used. Sapling was irrigated equally for approximately eight weeks (mid-March to mid-May). After eight weeks, water stress for saplings was imposed according to the field capacity (FC). Each pot was weighed at two-day intervals to detect the water loss in the pot and the amount of water reduced was given to the saplings according to the combinations of the treatment. Based on the FC, two different irrigation levels have been applied as non-stress and stress (100% and 50% of the FC, respectively). The start of the irrigation period is set to the level where 40% of the useful moisture is used in the potting soil. The treatments were divided into four main categories, including mineral solution at 0 (without Si or NO) i.e., control; Si: mineral solution + 10 mM Si; NO: mineral solution + 200 µM NO and Si + NO; mineral solution + 10 mM Si + 200 µM. In total, 8 different mineral solutions were performed for the plants under four main categories, which are Non-Stress + Control, Control + Stress, Si + NO n-Stress, Stress + Si, NO + Non-Stress, Stress + NO, Si + NO + Non-Stress, and Stress + Si + NO. In addition, solutions with concentrations of 10 mM Si and 200 µM NO were applied to sapling individually and in combination to reduce the effect of water deficit stress. Si and NO treatments were applied to each sapling plant every two weeks with 200 mL of nutrient solution (50% Hoagland) [16]. During the experiment, sapling plants were kept under natural conditions in the shade house.

### 2.2. Harvest in Saplings and Their Growth Parameters

Saplings were harvested at the end of the study (23 July), and the roots were then carefully removed from the pots. Then, root fresh weights (RFW), shoot fresh weight (SFW), shoot dry weight (SDW) and root dry weights (RDW) per sapling plant were performed. For dry weight in sapling, samples were dried at 65–70 °C for 48 h. The image J program measured the leaf area LA of the samples taken from 3 middle parts of the apical shoot of the sapling at harvest. Then, an average value was determined for each treatments and the LA of the saplings was detected. The areas of the leaves transferred to the image J program on the computer were calculated as cm^2^ [17]. To determine the change in relative shoot length (RSL) and relative shoot diameter (RSD) values were conducted at the beginning of water stress applications (15 May) and at the end of the trial (23 July). RSD and RSL were measured at the beginning of stress treatment in pots treated with water stress in mid-May and 23 July, in which 11 weeks of water stress was achieved. To detected RSD and RSL values were detected at the beginning and final water deficit stress treatments by the following formula [18].

Increment (%) = ((End value − Initial value) × End value^−1^)) × 100 (1)

### 2.3. Membrane Permeability (MP)

MP was assessed with the method specified by Lutts et al. [19]. Fully developed young leaves utilized 0.5 g of the sapling leaves selected by chance from all treatments was weighed, put into a tube and added 20 mL of distilled water, and then kept on hold at 40 °C for 30 min and the EC_1_ value of solution was determined. Then, the same solution was kept on hold at 100 °C for 10 min and the EC_2_ value of solution was detected. Membrane permeability values of the sapling leaves were calculated based on the following formula [19].

MP (%) = (EC_1_/EC_2_) × 100 (2)

### 2.4. Leaf Relative Water Content (LRWC)

LRWC values were conducted according to the method specified by Yamasaki and Dillenburg [20]. First, 1.5 cm diameter discs were removed from the samples taken from the 5th and 3rd leaves between the top and the middle part of the shoot of the sapling selected randomly from all treatment groups and their fresh weights were detected. The turgor weights of the leaf samples, which were kept on hold in water for 24 h, were measured and later, dried at 70 °C for 48 h in an oven up to a constant weight was arrived, and their dry weights were determined. Based on the data obtained, LRWC was calculated and the values were expressed as %. Turgor loss and leaf proportional water content of leaf were measured based on the following formula [20].

LRWC (%) = [(fresh weight − dry weight)/(turgor weight − dry weight)] × 100(3)

### 2.5. Stomatal Conductivity (SC)

SC was detected by the measurements performed between 12.00 and 14.00 h with the leaf porometer (Decagon Devices Inc., Model SC-1 Steady State Diffussion Porometer, Pullman, WA, USA) at the same positions of the 5th and 3rd leaves between the middle and peak part of the apical shoot of the sapling selected by chance from all application groups [21]. SC values is expressed in mmol m^−2^ s^−1^.

### 2.6. Chlorophyll Content (Chl)

The amount of chl in the leaves were determined by measuring with SPAD-502 Plus device (Konica Minolta Optics, Inc., Tokyo, Japan). The SPAD values were detected by two measurement values conducted at the same positions of the 3rd and 5th leaves between the middle and peak part of the apical shoot of the sapling randomly selected from all treatment groups. Then, the average of the SPAD readings were taken and the amount of chlorophyll was determined [22]. 

### 2.7. Leaf Temperature (LT), Proline Content and Lipid Peroxidation Analysis (MDA)

LT was detected with an infrared thermometer from the 5th and 3rd leaf parts of the plant randomly selected from the application groups on sunny days [23]. L-Proline values were measured in the leaves taken from the 3rd and 5th leaves between the middle and peak part of the apical shoot of the sapling according to the method of Bates et al. [24]. Samples (0.5 g) were ground in 10 mL of 3% sulfosalicylic acid to extract proline and the mixture was centrifuged at 10,000 rpm for 10 min. Then, 2 mL of the supernatant obtained from samples was added in tubes to which 2 mL of glacial acetic acid solution and 2 mL of the freshly prepared acid–ninhydrin were mixed. The tube was incubated in a water bath for 1 h at 90 °C for reaction, and afterward, in an ice bath, the reaction for samples was terminated. Then, samples were extracted by 5 mL toluene and, it vortexed for 15 s. Then standing at room temperature in the dark for at least 20 min to separate toluene and the aqueous phase, carefully the toluene phase was collected within these test tubes and the absorbance of the fractions for samples were read at 520 nm by a Shimadzu UV-1700. For the L-proline content in the leaves, the standard curve was prepared utilizing L-proline and results were detected as µg.g^−1^ leaf fresh weight.

MDA content of samples was detected as stated by Bolat et al. [8] utilizing the thiobarbituric acid analyses. Absorbance values of sample were recorded at 532 and 600 nm. MDA content in leaves was detected utilizing the following equation:

MDA (nmol/mL) = [(A532 − A600)/155000] × 106(4)

### 2.8. Mineral Analysis in Leaves

Leaf samples were then passed through pure water three times and dried with blotter paper. The leaves were kept in an oven at 65–70 °C for 48 h to dry. The leaves were ground in a porcelain mortar and conducted suitable for analysis. In the K, Ca and Mg atomic absorption spectrophotometer [25], the total N content of the leaves were detected as % by the modified Kjeldahl method [25]. In the samples made ready for analysis by applying dry burning, P was determined in the spectrophotometer device (UV-160 a Shimadzu) according to the vanadomolibdo phosphoric yellow color method, and the results were expressed as % [25].

### 2.9. Statistical Analysis

Analysis of data for all data was detected utilizing the SPSS software (SPSS Version 23, IBM, Herndon, VA, USA) average were determined by *Tukey’s* test. Differences for data at *p* ≤ 0.05 were considered significant. In addition, a *t*-test was performed by comparison between the stress and the non-stress irrigation applications in some morphological and physiological properties. The PCA was detected by JMP (JMP Version 13, SAS Institute Inc., Cary, NC, USA)). HCA (hierarchical clustering analysis) was detected utilizing a heatmap R package (R Version 3.6.3i, The R Foundation, Vienna, Austria).

## 3. Results

### 3.1. Plant Growth and Physiological Parameters

**Figure 1:**
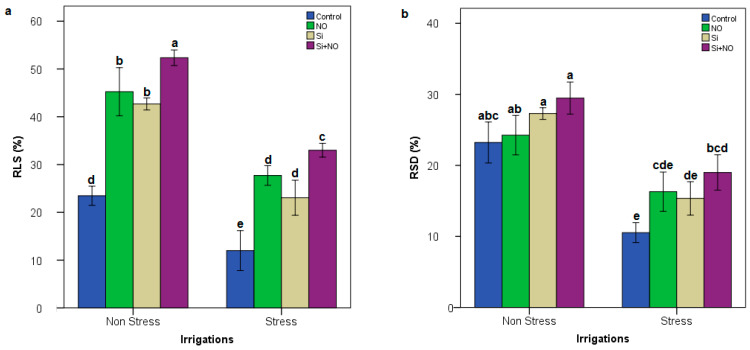
RSD and RSL in Milord apricot plant under different irrigation levels and treatments (**a**): Relative Shoot Length (RSL); (**b**): Relative Shoot Diameter (RSD) of apricot plant at different treatment and irrigation levels. Lowercase letters indicate significance differences (Tukey test; *p* ≤ 0.05) between treatments and irrigation levels.

Regarding our results, there were significant differences between both different irrigation levels and treatments, whereas there were no significant differences in their interactions exclude RSL and RSD. Average increase in RSL and RSD values ranged from 13.50 to 52.65%, from 10.37 to 29.64% in Stress + Control and Non-Stress + Si + NO application, respectively. Considering the effects of stress on both RSL and RSD parameters were examined in Milord apricot plants, the highest values were determined in the Non-Stress + Si + NO application, while the lowest values were obtained from the Control + Stress application (Figure 1). Water stress led to decreases in SFW, RFW, SDW, RDW and LA in Milord apricot plant. SFW, SDW, RFW, RDW and LA ranged from 108.35 to 168.57 g, from 51.01 to 88.92 g, from 88.04 to 153.50 g, from 38.68 to 43.61 g and from 41.76 to 62.03 (cm^2^) in Stress and Non-stress, respectively (Table 1).

There were significant differences between both different irrigation levels and treatments, whereas there were no significant differences in interactions of SC, Chl and LRWC. The SC, MP, Chl, LRWC and LT ranged from 49.41 to 112.96 (mmol m^−2^ s^−1^), from 19.83 to 15.01%, from 38.83 to 43.47, from 73.32 to 79.30 (%) and from 34.57 to 31.92 (°C) for stress and non-stress, respectively. Among irrigation levels, the highest values of SC, Chl and LRWC were determined in Non-stress, while the lowest value was obtained in the Stress condition. Among the treatments, the highest values of SC, Chl and LRWC were determined in Si + NO, while the lowest values were detected in the Control + Stress treatment condition. On the other hand, the opposite occurred in the effects of applications and water stress on MP and LT (Table 2).

### 3.2. Biochemical Parameters

**Figure 2:**
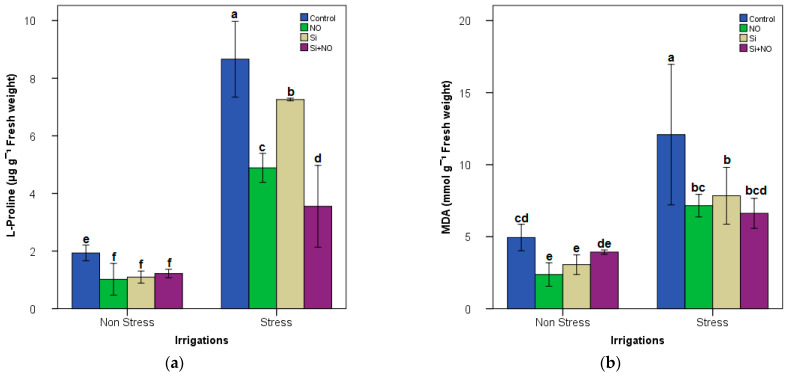
L-Proline (**a**) and MDA (**b**) contents in Milord apricot plant under different irrigation levels and treatments. Lowercase letters indicate significance differences (Tukey test; *p* ≤ 0.05) between treatments and irrigation levels.

Considering biochemical parameters, L-Proline and MDA did show significant differences from the water stress. The interaction between water stress and Si and NO treatments on leaf L-Proline and MDA values were significant. L-Proline and MDA increased at water stress conditions in Milord apricot plants. L-Proline in Milord apricot plants ranged from 9.04 to 1.10 (µg g^−1^) in Stress and Non-Stress + NO, respectively (Figure 2a). MDA in Milord apricot plants ranged from 11.66 to 2.83 (mmol g^−1^) in Stress and Non-Stress + NO, respectively (Figure 2b). 

**Figure 3:**
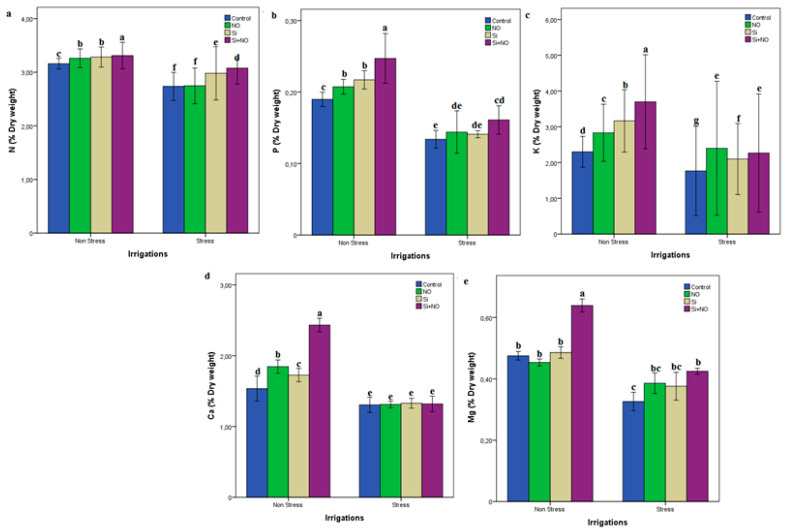
Macro element contents in Milord apricot plant under different irrigation levels and treatments. Lowercase letters indicate significance differences (Tukey test; *p* ≤ 0.05) between treatments and irrigation levels, K: Potassium (**c**), N: Nitrogen (**a**), P: Phosphorus (**b**), Ca: Calcium (**d**), Mg: Magnesium (**e**).

While the highest values of L-Proline and MDA were determined from Stress + Control conditions, the lowest values were obtained from applications under Non-Stress + NO conditions (Figure 2 and Figure 3). Water stress caused significant reductions in P, N, K, Mg and Ca leaf in Milord apricot plants. On the other hand, NO, Si and NO + Si applications in Non-Stress and Stress conditions generally led to an increase in mineral content. P, N, K, Mg and Ca values ranged from 2.73 to 3.31%, from 0.13 to 0.25%, from 2.3 to 3.0%, from 1.3 to 2.4% and from 0.32 to 0.63% in Stress and Non-Stress treatments, respectively (Figure 3).

### 3.3. Multivariate Data Analysis 

**Figure 4:**
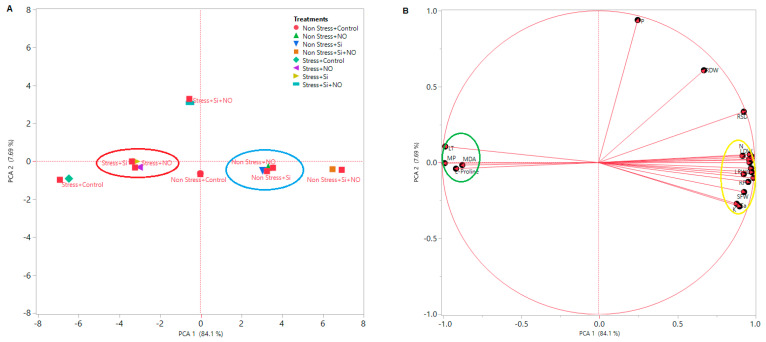
The loading plot of all variables represented in PCA for biochemical, physiological, morphological and macronutrient elements in apricot plant. Different irrigation levels and treatments in PCA are shape-A (**A**), morphological, biochemical and macronutrient parameters shape-B (**B**). LT: Leaf Temperature, MP: Membrane Permeability, LRWC: Leaf Relative Water Content, RFW: Root Fresh Weight, Chl: Chlorophyll, SDW: Shoot Dry Weight, SFW: Shoot Fresh Weight, Leaf Area, RDW: Root Dry Weight, LA: SC: Stomatal Conductive, RSL: Relatif Shoot Leight, RSD: Relatif Shoot Diameter, P: Phosphorus, K: Potassium, Mg: Magnesium, N: Nitrogen, Ca: Calcium. Considering Figure 5, cluster-I; stress + control, cluster-II; Stress + Si and Stress + no, cluster-III; Non-Stress + Control, Stress + Si + NO and Stress + Si + NO, cluster-IV; Non-Stress + Si + NO, Non-Stress + Si and Non-Stress + NO. It was found that applications such as Si or NO to the stress group had a healing effect on some morphological and physiological parameters by bringing them closer to the Non-Stress + Control. Cluster I consists of the Stress + Control group. Cluster II shows that Stress + Si and Stress + NO had the same positive effect. Cluster III contains the Non-Stress + Control group and the Stress + Si + NO group. Thus, it seen that the Si and NO combination alleviate effect and almost approached the non-stress + control group. Cluster IV includes Non-Stress + NO, Non-Stress + Si and Non-Stress + Si + NO. On the other hand, MP and LT are in cluster-A; cluster-B contains L-Proline and MDA, cluster-C contains K and Ca; and cluster-D includes RDW, P, Mg, SDW, SFW, RFW, SC, Chl, N, LRWC, LA, RSL and RSD (Figure 4). In Stress, the MP, LT, L-Proline and MDA values increase and the red color on the heat map is highest for Stress + Control, while the lowest MP and LT values are obtained at Non-Stress + Si + NO. The lowest L-Proline and MDA values were obtained from Non-Stress + NO. When HCA is examined, the lowest values of LRWC, SC, RSL, RSD, LA, Chl, SFW, SDW, RFW, RDW, N, P and Mg values are located at stress irrigation level in the control and are indicated in dark blue. The highest LRWC, SC, RSL, RSD, LA, Chl, SFW, SDW, RFW, RDW and N values are in the Non-Stress + Si + NO treatment and are shown in red (Figure 5).

PCA provides a graph that simplifies the visualization and understanding of the dataset and variables. To analyze the morphological and physiological properties of NO, Si and Si + NO treatments under water stress, basically two different PCA were conducted and named as B-shaped and A-shaped PCA, respectively. These analysis factors have different parameters (to extract and show relationships between LRWC, SC, RSL, RSD, LA, Chl, MP, LT, L-Proline, MDA, SFW, SDW, RFW, RDW, N, P, K, Ca and Mg). Regarding Figure 4A, Non-Stress + Si + NO, NO + Non-Stress and Si + NO n-Stress are very close to each other and are in the same group, while Non-Stress + Control forms a single group. In addition, Non-Stress + Control is less effective than other treatments, but its effect is positive compared to stress irrigation level. Similarly, Stress + Si and Stress + NO are in the same group, and the treatment of Stress + Si + NO shows a better effect than the treatment of Si and NO alone, forming a separate group. This also indicates a positive relationship between water stresses and alleviate treatments. In other words, the negative effect of water stress has decreased with alleviate practices. Based on Figure 4B, there is a positive relationship between LRWC, SC, RSL, LA, Chl, RDW, RSD, SFW, SDW, RFW, P, N, K, Mg and Ca. Therefore, as the amount of irrigation increased (from Stress to Non-Stress), the values of the above parameters also increased. On the other hand, there was a negative correlation among L-Proline, MP, MDA and LT parameters in different irrigations and treatments, and MP, MDA, LT and L-Proline content increased as the amount of water decreases (from Stress to Non-Stress).

## 4. Discussion

Although significant development has been noted in comprehending the response of some plant species to water deficit by applying NO and Si in the last few decades [7,8], one question remains obscure among scientists: How can one detect the reactions of plants under potting environments to correctly predict field responses after the Si + NO interactive application? Indeed, despite much recent research regarding the effects of Si and NO alone on water-deficit stressed plants, the mechanisms underlying Si + NO interactive application effects are not well understood in Milord apricot and many other plant species. Milord apricot were, therefore, evaluated under non-stress and water deficit stress conditions to screen them for water stress tolerance and to notice if a difference in their physiological and biochemical responses to water deficit stress exists between the stress conditions and applications by applying NO, Si and their combination together (Si + NO). Our findings indicated that most of the biochemical and physiological parameters of the Milord apricot were affected by NO, Si and NO + Si treatments under water stress and non-stress conditions. In our study, SFW, SDW, RFW, RDW, RSL, RSD, LA, SC and LRWC declined under water stress (Table 1 and Table 2), which might be because, apart from affecting the plant’s relationship with water, water stress causes the closure of stomata to decrease water loss transpiration by affecting physiological processes. This can be explained by the fact that water stress decreases growth (e.g., expansion and elongation growth as described by Shao et al., [26]), owing to the reduction in cell division and enlargement, cell metabolic activities and, consequently, dry weight of plant, which reflects in growth parameters in apricot. Indeed, the relative water content in the leaf area decreases, which directly affects other vital events such as the photosynthesis of plants [26]. Water stress causes a decrease in SC and LRWC ratios and an increase in LT values (Table 2), whereby some physiological processes such as closure of stomata causes a decrease in the level of CO_2_ in the leaf tissues and also increase in superoxide radicals, which are called oxygen species [27]. Radicals such as hydroxide radicals and hydrogen peroxide cause lipid peroxidation, followed by reduction of pigments, reduction of membrane and structure of proteins. They play a key role in susceptibility and tolerance to water stress. Considering the parameters obtained from water stress studies conducted by different researchers, they stated that water stress negatively affects plant development and growth [28,29,30,31,32,33].

Based on our findings, NO and Si treatments applied to decrease the negative effect of water stress have positive results on morphological parameters. Many authors have indicated a remarkable improvement in various plant growth parameters with NO and Si treatment in many crop plants such as wheat, cowpea, maize, Arabidopsis, strawberry, and pea under water stress conditions [2,7,8]. In addition, morphological parameters have also observed when Si and NO are given separately or in combination to plants under water stress. Dehghanipoodeh et al. [34] noted that water stress in strawberry reduced plant dry and fresh weights, plant growth and leaf area, while Si application positively affected plant biomass production under stress. Additionally, Nabi et al. [35] reported that NO plays an important role in the development and growth of the plant in stress factors, namely, drought stress and some adaptation mechanisms (change of the root system so that the plants can obtain more water, and permeability of the cuticle in the leaves). Furthermore, NO causes leaves to curl, reducing water loss by evaporation, and thus a reduction in stomata size [36]. On the other hand, water stress in our findings declined L-RWC, SC and Chl values, which subjects the cell membranes for rootstock to changes including penetrability and reduce in sustainability, probably due to the ability to osmotic adjustment due to the increased content of L-proline and MDA (Figure 2a,b and Table 2), similar to results of some other reports [37,38,39]. It is, indeed, documented that proline protected plants by stabilizing enzymes against various stresses, proteins, membranes and the mitochondrial electron transport complex II [1,8]. Besides this, NO emerges as a vital element in drought tolerance of different plants by improving L-Proline, MDA and membrane permeability. In addition, NO is an active factor by promoting signaling in some pathways (such as closure of stomata by sending a signal to ABA to prevent water loss) [37]. According to Biju et al. [3], Si in lentils under drought stress increases RWC by helping lentils take up water, regulates the water relationship in the cell, adjusts membrane permeability in balancing ions and improves chlorophyll pigment content by providing assimilate accumulation to developing tissues [39]. 

During the metabolite activities of plants, the uptake of some nutrients in plant cells is important. In our current study, however, P, N, K, Mg and Ca uptake of plants was restricted under water stress conditions (Figure 3), which are consistent with the findings noted by Dehghanipoodeh et al. [34]. Besides this, a decrease in the amount of K, Na, Mg, Ca, B, Zn and Fe were observed with the increase in the severity of water stress in Myrobolan 29 C and Garnem rootstock under in-vitro conditions [40], which supports our findings. NO was determined in that the N and K concentrations of the Si and NO applied from the soil in a single and combined way significantly affected the plant. Similar reports were also detected in wheat where NO is reported to increase N and K under water stress [41]. In addition, the studies of Ramos Artuso et al. [42] and Zhu et al. [10] gave similar findings with our study, and the use of NO and its donors activates acid phosphates in root tissues and facilitates their uptake. On the other hand, the researchers found that Si and NO were capable of modifying the root structure [36]. Majeed et al. [43] noted that NO and its donors have a positive effect on plant nutrients and activate the root zone in the uptake of minerals in their drought study on wheat. Si has important effects on the development and growth at different stages in the life cycle of the plant, from reducing transpiration, reducing antioxidant defense systems to the development of photosynthetic activity [44].

## 5. Conclusions

In present study, we expanded upon our previous results concerning the physio-biochemical attributes of NO, Si and Si + NO interactive treatments under water deficit stress in some Prunus species like Mliord apricot. Here, we also present findings on the potential capability of NO, Si and Si + NO treatments to enhance Milord apricot fitness to withstand problematic conditions such as water deficit stress, through multiple possible adverse factors that indirectly or directly result in a better defense strategy to make use of the available water under these conditions. Based on our results, water stress caused a strongly decreasing effect in relative water content of the leaf and gas exchange of leaf stomata. Indeed, significant increases in MP, MDA and L-Proline were observed under stress conditions. Si and NO applications were positive in SFW, SDW, RFW, RDW and LA. On the other hand, Si and NO significantly reduced MP, MDA and L-Proline, and was effective in LWRC, SC, N, P, K, Ca and Mg. Overall, these findings highlight that these applications, especially the Si + NO interactive application, could be considered as a strategy to improve the quantity, quality and yield of many plant species in water deficit stress conditions. Further studies are needed to show whether these findings may be predicted to other tree deciduous species both in the same applications and under the same conditions.

## Figures and Tables

**Figure 5:**
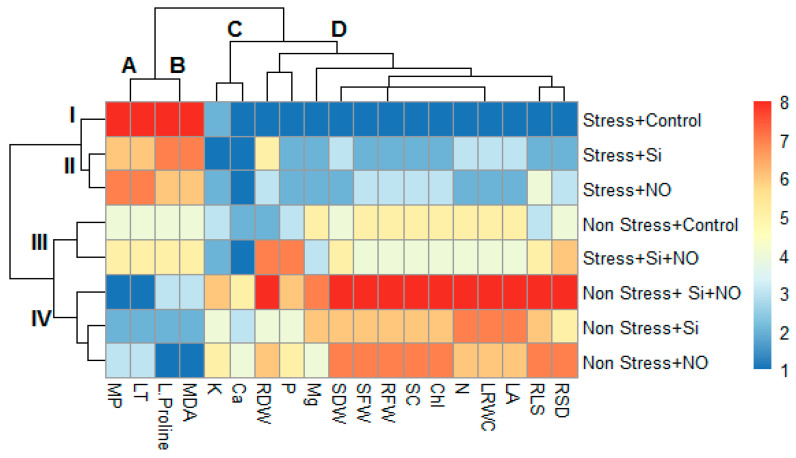
HCA (hierarchical clustering analysis) for biochemical, physiological, morphological and macronutrient parameters in apricot plant. RFW: Root Fresh Weight, SDW: Shoot Dry Weight, LT: Leaf Temperature, MP: Membrane Permeability, SFW: Shoot Fresh Weight; LRWC: Leaf Relative Water Content, RDW: Root Dry Weight, LA: Leaf Area, Chl: Chlorophyll, SC: Stomatal Conductive, RSD: Relative Shoot Diameter, RSL: Relative Shoot Length, K: Potassium, P: Phosphorus, N: Nitrogen Mg: Magnesium, Ca: Calcium. A, B, C and D represent the groups of parameters studied. I, II, III and IV represent the groups of the treatments studied.

**Table 1:** Some morphological properties detected at harvest in leaves of Milord apricot variety subjected to chemicals applications and different irrigation regimes.

Irrigation Level	SFW (g/Plant)	SDW (g/Plant)	RFW (g/Plant)	RDW (g/Plant)	LA (cm^2^)
Non-Stress	168.57 a	88.92 a	153.50 a	43.61 a	62.03 a
Stress	108.35 b	51.01 b	88.04 b	39.68 b	41.76 b
*t*-test	*	*	*	*	*
Treatments					
Control	110.80 d	52.15 c	89.41 c	28.47 c	40.11 b
Si	137.04 c	70.61 b	122.05 b	40.92 b	56.03 a
NO	142.50 b	73.97 b	123.82 b	40.20 b	51.48 a
Si + NO	163.70 a	83.13 a	147.80 a	56.99 a	59.96 a
Tukey test	*	*	*	*	*

*: Significance level at *p* ≤ 0.05 was detected for the irrigation level (*t*-test) and treatment (Tukey test). Different letters in columns represent statistical differences. SFW: Shoot Fresh Weight, LA: Leaf Area, RDW: Shoot Dry Weight, RFW: Root Dry Weight, SDW: Root Fresh Weight.

**Table 2:** Effects of water stress and solutions on some physiological properties in Milord apricot variety.

Irrigation Level	SC (mmol m^−2^ s^−1^)	MP (%)	Chl	LRWC (%)	LT (°C)
Non Stress	112.96 a	15.01 b	43.47 a	79.30 a	31.92 b
Stress	49.41 b	19.83 a	38.83 b	73.32 b	34.57 a
*t*-test	*	*	*	*	*
Treatments					
Control	61.85 d	19.80 a	38.53 c	73.26 d	34.63 a
Si	79.57 c	16.85 c	42.40 b	77.32 b	32.87 c
NO	87.12 b	17.73 b	42.40 b	75.80 c	33.32 b
Si + NO	96.19 a	15.32 d	43.05 a	78.86 a	32.15 c
Tukey test	*	*	*	*	*

*: Significance level at *p* ≤ 0.05 was detected for the irrigation level (*t*-test) and treatment (Tukey test). Different letters in columns represent statistical differences. SC: Stomatal conductivity, MP: Membrane Permeability, Chl: Chlorophyll Content, LRWC: Leaf Relative Water Content, LT: Leaf Temperature

## Data Availability

Not applicable.
